# Community Composition and Diversity of β-Glucosidase Genes in Soils by Amplicon Sequence Variant Analysis

**DOI:** 10.3390/genes16080900

**Published:** 2025-07-28

**Authors:** Luis Jimenez

**Affiliations:** Biology and Horticulture Department, Bergen Community College, 400 Paramus Road, Paramus, NJ 07652, USA; ljimenez@bergen.edu

**Keywords:** cellulose, cellulases, β-glucosidase genes, bacteria, mold, soil, next generation sequencing, amplicon sequence variants

## Abstract

Cellulose, the most abundant organic polymer in soil, is degraded by the action of microbial communities. Cellulolytic taxa are widespread in soils, enhancing the biodegradation of cellulose by the synergistic action of different cellulase enzymes. β-glucosidases are the last enzymes responsible for the degradation of cellulose by producing glucose from the conversion of the disaccharide cellobiose. Different soils from the states of Delaware, Maryland, New Jersey, and New York were analyzed by direct DNA extraction, PCR analysis, and next generation sequencing of amplicon sequences coding for β-glucosidase genes. To determine the community structure and diversity of microorganisms carrying β-glucosidase genes, amplicon sequence variant analysis was performed. Results showed that the majority of β-glucosidase genes did not match any known phylum or genera with an average of 84% of sequences identified as unclassified. The forest soil sample from New York showed the highest value with 95.62%. When identification was possible, the bacterial phyla Pseudomonadota, Actinomycetota, and Chloroflexota were found to be dominant microorganisms with β-glucosidase genes in soils. The Delaware soil showed the highest diversity with phyla and genera showing the presence of β-glucosidase gene sequences in bacteria, fungi, and plants. However, the Chloroflexota genus *Kallotanue* was detected in 3 out of the 4 soil locations. When phylogenetic analysis of unclassified β-glucosidase genes was completed, most sequences aligned with the Chloroflexota genus *Kallotenue* and the Pseudomonadota species *Sphingomonas paucimobilis*. Since most sequences did not match known phyla, there is tremendous potential to discover new enzymes for possible biotechnological and pharmaceutical applications.

## 1. Introduction

Soils are the largest reservoirs of organic carbon and microorganisms have a fundamental role in carbon cycling by decomposing complex organic polymers such as lignin, chitin, and cellulose [1,2,3,4,5,6]. The decomposition of soil organic matter (SOC) provides chemical compounds that can be used by the dynamic microbial, plant, and animal communities present in soils. Without the action of these microbial communities, these complex organic polymers cannot be used as nutritional sources by other organisms. However, soil microbial activity is affected by different biotic and abiotic factors present in soil such as carbon content, land management, climate, temperature, predation, parasitism, competition, etc., but pH has been shown to be the most important factor affecting community composition and diversity [2,4,5].

The great majority of microorganisms in the environment are unculturable presenting a difficult challenge to assess their biochemical contributions to different processes in soils [4,6]. However, direct analyses of 16S rRNA and internal transcribed spacer (ITS) genes from soil samples have provided a better understanding of microbial community structure and distribution but do not reveal any information about the function of microorganisms and their roles in the cycle of organic and inorganic compounds [2,4,6]. Microbial communities in soils worldwide are dominated by bacteria belonging to the phyla Pseudomonadota, Actinomycetota, Acidobacteriota, Bacteroidota, and Chloroflexota [2,4,5]. Fungi communities are comprised mostly of the phyla Ascomycota and Basidiomycota [6].

To determine the contribution of microbial populations to different biochemical processes taking place in soils, functional genes have been studied around the world [7,8,9,10]. For instance, the distribution and diversity of ammonia oxidizers, nitrate reducers, cellulose degraders, and photosynthetic bacteria in soils have provided important information of the community structure and diversity of functional groups [11,12,13,14,15,16]. This analysis has been expanded to industrial processes such as bioremediation and anaerobic bioreactors where processes were optimized by describing and recognizing the contribution of specific bacterial populations with functional genes critical to operational success [17,18,19].

However, the activities of these bacterial functional groups are not limited to specific bacterial phyla, genera, or species [14,16,20,21]. In some processes functionality is found through different phyla, genera, and species. However, there are situations where functional groups represent a small fraction of the microbial community [11,13,14,22]. Genetic sequences coding for specific enzymes can be different but the activity is the same. An example of this are the genetic sequences coding for cellulases in soils [14,15,20,21,22]. Cellulases degrade cellulose to glucose which is used by other organisms to complete different metabolic processes [23]. Furthermore, in soils, cellulose is the most important carbon source for microbial respiration [1,5]. Several microorganisms are known to be cellulolytic such as *Streptomyces* sp., *Massilia violaceinigra*, *Bacteroides cellulosolvens*, *Clostridium* sp., *Irpex lacteus*, *Cellulomonas* sp., etc. [14,15,18,20,24,25]. Recent studies have shown that even several bacterial species obtaining their carbon from cellulose were previously not recognized as cellulolytic [14,15,22].

Cellulose, a polymer of β-1,4- linked glucosyl residues, is the most abundant complex organic polymer in soils accounting for approximately 45% of the photosynthetic products [1]. The biodegradation of cellulose to glucose is a synergistic process based upon the activity of three different enzymes named glycoside hydrolases (GH) [21,23]. Therefore, complete degradation is rarely performed by a single microorganism but rather requires a community of organisms to complete the process [1,15,21]. These three enzymes show different substrate specificity degrading glycosylic bonds present between carbohydrates or carbohydrates and other non-carbohydrate moieties. The first enzymes, e.g., endoglucanases, cut inside the cellulose structure. This is followed by a second enzyme called exoglucanase which cuts the external residues created to produce cellobiose. The last enzyme, β-glucosidase hydrolyzes cellobiose to simple sugars such as glucose. Endo and exoglucanases are inhibited by cellobiose. However, β-glucosidase is often inhibited by its product glucose. Therefore, β-glucosidase is the rate-limiting enzyme during cellulose biodegradation [24,25]. Maintaining a high hydrolysis rate of cellulose will require efficient β-glucosidases tolerating high levels of glucose. It has been shown that the activity of β-glucosidases in soils control the mineralization of SOC [16,26,27]. Furthermore, these enzymes play vital roles in other biological processes such as terpenols and flavonoid production from glycoside precursors [28]. The transglycosylation activity of some β-glucosidase enzymes makes them potentially useful to industrial processes and pharmaceutical applications.

Because of their specificity, GH genes belong to different functional families [14,21,23]. For example, GH5 genes are associated with endoglucanase activity. GH7 enzymes are fungi-specific while GH6 genes are found in bacteria and fungi [29]. Both groups exhibit endoglucanase and exoglucanase activity. GH48 genes are predominantly shown to be associated with exoglucanase activity and degrade crystalline cellulose [14]. Families GH1 and GH3 genes are mostly related to β-glucosidases found in bacteria, fungi, plants, and animals [21,24,25,30].

GH1 enzymes have been shown to be more tolerant to glucose. However, few studies have ascertained the distribution and identity of β-glucosidase genes in soils. In forest soils, β-glucosidase activity increased with organic matter content with no seasonal differences in enzyme activity [26]. Recent studies have been reported on the diversity of β-glucosidase genes in semiarid European soils in Spain and temperate soils in the state of New Jersey, USA [24,31]. PCR analysis and cloning libraries of β-glucosidase genes extracted from those soils showed that the predominant bacterial communities were led by the Pseudomonadota genera *Alteromonas* and *Rhodanobacter*, respectively. Himalayan soil samples have been used to develop cloning libraries of β-glucosidase genes with bacteria belonging to the phylum Pseudomonadota as the dominant types [30]. However, bacteria belonging to the Bacteroidetes were dominant in compost and cow dung samples. The soil sample showed higher β-glucosidase genes diversity than the other samples analyzed.

PCR and denaturing gradient gel electrophoresis (DGGE)-based community analysis has reported the presence of five bacterial phyla with β-glucosidase genes in compost samples [25]. Most of the bacterial sequences were related to the phyla Pseudomonadota, Bacillota, Actinomycetota, and Bacteroidota. Fungi β-glucosidase genes detected were predominantly Ascomycota and Basidiomycota with the genus *Aspergillus* as the most common taxa. Different soils from the states of New Jersey and New York in the USA have shown that β-glucosidase genes were the most abundant GH genes with a reported 100% frequency [15]. Cloning libraries demonstrated that the dominant community was composed of Actinomycetota species belonging to *Nakamurella multipartita* and different types of *Streptomyces*. However, 40% of the sequences were reported to be unclassified.

Although PCR analysis and cloning libraries of β-glucosidase genes from soils provide a better understanding of the microbial population genetic potential than culture studies, a limited number of clones were analyzed in all the above studies, leading to an underestimation of the β-glucosidase genetic potential in soils. PCR and cloning can also introduce bias, hindering the detection of genetic sequences not amplified by the selected primer pair. Furthermore, the depth of the sequencing reactions is extremely limited because the low numbers of reads per sample when individual reads are analyzed by Sanger sequencing. Because of the limited depth, low abundance taxa can go undetected and the composition and diversity of the microbial community containing β-glucosidase genes can be severely underestimated [24,31]. Random sequencing of clone libraries can also be time-consuming, expensive, inaccurate, and labor-intensive.

Next generation sequencing analysis has significantly increased the numbers of β-glucosidase gene sequences analyzed in coniferous forest soils from the Bohemian Forest mountain range in Central Europe [26]. High throughput analysis allowed the simultaneous sequencing of complex communities resulting in an increase in depth, accuracy, and resolution. Fungal and bacterial sequences belonging to GH 1 and 3 genes were the most abundant cellulases detected. Fungi from the Ascomycota and Basidiomycota were the predominant taxa. However, unclassified sequences ranged from 2 to 50%. Bacterial GH1 genes were mostly related to the phyla Pseudomonadota and Actinomycetota while GH3 was dominated by Bacillota and Acidobacteriota. Unclassified β-glucosidase sequences for both GH1 and GH3 genes ranged from 1 to 72%.

In other studies, the land management of soils in a citrus plantation in China have led to the reduction in GH3 β-glucosidase genes and lower bacterial diversity [32]. Community composition was significantly altered with forest soils showing a higher abundance of Pseudomonadota than citrus soils. On the other hand, the Acidobacteriota which showed the second highest numbers in citrus soils were detected in significantly higher numbers in forest soils. Fungi communities with GH3 genes were prevalently composed of Ascomycota fungi. SOC was reported to be the most important factor influencing β-glucosidase activity and GH genes in soils.

Subtropical forest soils from China have been analyzed by metagenomic analyses and results demonstrated that GH1 and GH3 were the dominant genes for plant decomposition with depth as a positive factor increasing their abundance [33]. GH1 and GH3 genes were significantly correlated to the mineralization of SOC. The dominant phyla consisted of Actinomycetota, Acidobacteriota, Pseudomonadota, and Chloroflexota. On the other hand, fungi contribution to the β-glucosidase gene community was more significant than bacteria in Italian soils [27]. Gene distribution correlated with SOC. Fungal β-glucosidase gene abundance was a better indicator than any other GH genes of SOC in soils. Furthermore, gene presence in fungi correlated with the corresponding enzyme activity. However, the presence of bacterial β-glucosidase genes in soils did not. Agricultural soils in Iowa, USA, have been analyzed using metagenomic and enzymatic activity analysis indicating that β-glucosidase were the most abundant GH genes in fertilized prairie soil followed by endoglucanases genes [16]. The Actinomycetota and Pseudomonadota were the dominant taxa with β-glucosidase genes. Soils from tea plantations in China have demonstrated a significant increase in β-glucosidase genes after nitrogen (N) application. The absolute abundance of the genes was increased with increasing N rates [34]. The predominant taxa carrying β-glucosidase genes were members of the Acidobacteriota, Pseudomonadota, Actinomycetota, Bacteroidota, and Chloroflexota. At the genus level the four highest relative abundances reported were *Silvibacterium*, *Acidobacterium*, *Streptomyces*, and *Rhodanobacter*.

Recent studies in soils from the Northeastern part of the USA have shown that similarity between bacterial communities in soils decreased with increasing distance, indicating the dispersal limitations of some taxa to colonize different habitats. However, deterministic and stochastic factors may have affected the biogeographical distribution of the different bacterial phyla and genera. All soils were shown to be predominantly composed of different relative abundance values by bacteria belonging to the phyla Pseudomonadota, Actinomycetota, Acidobacteriota, Chloroflexota, and Bacteroidota [35]. Bacteria from the genera *Bradyrhizobium* and *Rhodoplanes* were the dominant taxa in all soils. However, the functionality of the different bacteria was difficult to determine because analyses were based upon 16S rRNA sequencing.

The distribution and community structure of bacteria with β-glucosidase genes have been previously studied in soils from the states of New Jersey (NJ) and New York (NY) [15,31]. However, community composition and diversity were based upon PCR analysis and cloning libraries with a limited number of sequences which might have underestimated the genetic potential of soils containing β-glucosidase genes. The major objective of this study was to use next generation sequencing of β-glucosidase genes, e.g., GH1 genes, detected in soil samples from the states of Delaware (D), Maryland (M), NJ, and NY to determine the genetic potential to degrade cellobiose and convert it to glucose increasing the cycling of carbon in soils. Who are the phyla or genera carrying these genes in soils? What is the genetic structure similarity of the populations carrying β-glucosidase genes between soil locations? What is the genetic potential of each soil to degrade cellobiose to glucose?

## 2. Materials and Methods

### 2.1. Soil Sampling

Surface soil samples were aseptically collected as previously described [35]. The first sample was obtained in the town of Fair Lawn in the state of NJ. Two other samples were taken at different locations, e.g., 100 m away, from interstate highway 95 North (I-95 N). I-95 N is the main north–south highway connecting southern and northern states and major cities of the eastern seaboard of USA. One sample was taken in the state of D and another one in the state of M. A fourth sample came from the state of NY, e.g., undisturbed conifer soil, at the Balsam Lake Mountain Wild Forest in the Catskill Mountains, Hardenburgh, NY (elevation 3700 feet).

### 2.2. DNA Extraction and PCR Amplification of β-Glucosidase Genes in Soils

Microbial DNA was extracted from duplicate soils from each state using the ZR Soil Microbe DNA MiniPrep Protocol (Zymo Research, Irvine, CA, USA) as previously described [36]. DNA concentration was determined by using the Qubit^®^ dsDNA HS assay as described by Jimenez et al. [37]. To analyze the presence of beta-glucosidase genes in soils, PCR amplification was performed using degenerate primers BGH1F (CCTACCAGATYGARGG) and BGHF1R (GAGGAAGRTCCCARTG), that amplified a 300-base pair (bp) fragment [25]. Reaction conditions were as follows: 94 °C for 5 min, followed by 35 cycles consisting of denaturation at 94 °C for 1 min, annealing at 55 °C for 1 min, and extension at 72 °C for 1 min. After the 35 cycles were completed, a final extension step at 72 °C for 10 min was added to the reaction.

Ready-To-Go (RTG) PCR beads (GE Healthcare, Buckinghamshire, UK) were used for each PCR reaction volume as previously described [35]. Reaction mixtures were added to a T100TM thermal cycler (Bio-Rad Laboratories, Hercules, CA, USA) or Mastercycler thermal cycler (Eppendorf Scientific, Westbury, NY, USA). After PCR amplification, amplicon detection was analyzed by gel electrophoresis using the FlashGel system (Lonza Inc., Rockland, ME, USA) as described by Jimenez et al. [37]. A FlashGel DNA Marker (Lonza Inc., Rockland, ME, USA) with fragment sizes ranging from 100 bp to 4 kilo bases (kb) was used to determine the presence of the correct DNA fragments.

### 2.3. Next Generation Sequencing Analysis of β-Glucosidase Genes in Soils

To determine the identity of β-glucosidase genes in soils, next generation amplicon sequencing of the 300 pb DNA fragment was performed by Azenta Life Sciences (South Plainfield, NJ, USA) using an Illumina Miseq protocol (Illumina, San Diego, CA, USA). Pair-end analysis was performed by using the following non-degenerate DNA primer pairs, pair 1F CCT ACC AGA TCG AAG G and 1R GAG GAA GAT CCC AAT G, pair 2F CCT ACC AGA TTG AGG G and 2R GAG GAA GGT CCC AGT G.

Amplicon sequence variants (ASV) were clustered at 100% similarity [38]. The top 10 sequences at the top, middle, and end of the file organized numerically from the highest to lowest abundance numbers were analyzed using the GenBank server of the National Center for Biotechnology Information (NCBI; http://blast.ncbi.nlm.nih.gov/Blast.cgi, accessed on 28 March 2025) and the BLAST (blastn) algorithm [39]. Sequences that did not match any β-glucosidase genes were discarded and replaced with the next numerical sequence. Venn diagrams were calculated as described by Behnke-Borowczyk et al. [40]. The Jaccard similarity index was calculated as described by Real and Vargas [41].

### 2.4. Phylogenetic Analysis of Unclassified β-Glucosidase Genes

Multiple sequence alignment and phylogenetic analysis of β-glucosidase gene sequences was performed using Phylogeny.fr (http://phylogeny.lirmm.fr/phylo_cgi/simple_phylogeny.cgi (accessed on 27 June 2025) as described by Dereeper et al. [42]. The pipeline used MUSCLE, Gblocks, PhyML, and TreeDyn to develop a phylogenetic analysis of a given set of unaligned sequences [42]. 

The top two most abundant unclassified sequences from each soil sample were analyzed and compared to sequences detected in this study identified as *Thermomonospora amylolytica*, *Kallotenue* sp., *S. paucimobilis*, *Cellulomonas biazotea*, and *Agrobacterium tumefaciens* (Table 1). These bacterial sequences were selected based upon either being the most abundant sequences found in each soil or that they were detected at least in two locations.

## 3. Results

### 3.1. β-Glucosidase Genes Soil Communities Based upon Phyla

**Figure 1:**
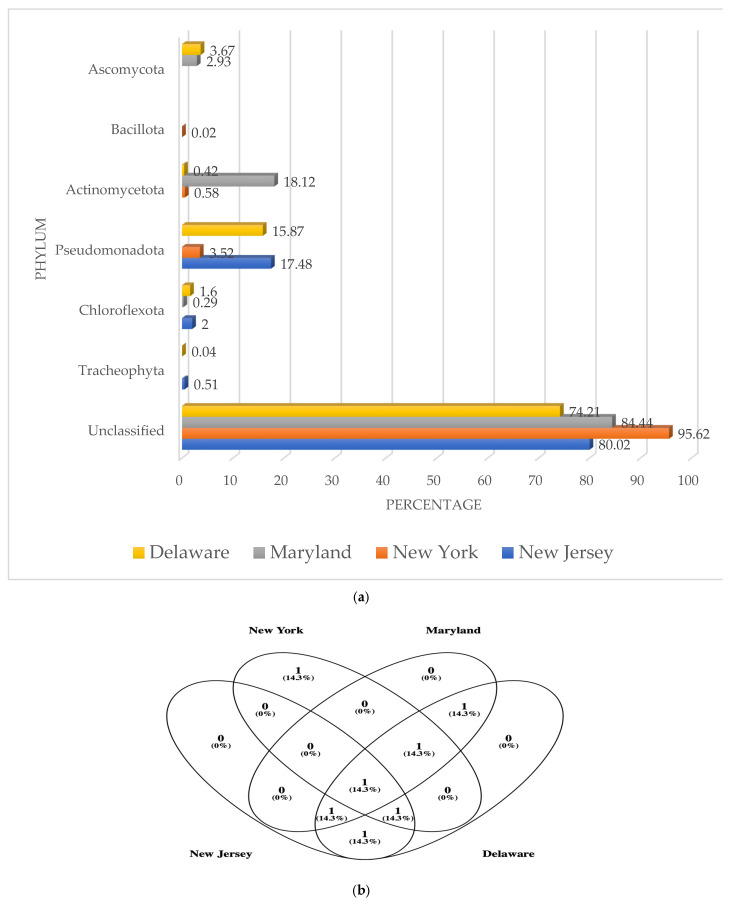
(**a**) Percentage of dominant phyla with β-glucosidase genes; (**b**) Ven diagram of common phyla.

An average of 2298 DNA sequences per soil were analyzed for a total of 9192. The NY (5204) sample showed the highest number of genetic sequences that were validated after BLAST analysis (version 2.16.0) to be coding for β-glucosidase genes followed by D (2369), NJ (1196), and M (423). The numbers of total phyla with β-glucosidase genes in all four soil locations ranged from 3 to 5. Figure 1a shows that most β-glucosidase genes were unclassified DNA sequences that did not match any known phylum. The highest number of unclassified sequences were detected in the NY soil with 95.62% followed by M (84.44%), NJ (80.02%), and D (74.21%).

The D soil showed the highest diversity of β-glucosidase genes with five different phyla detected. The highest frequency was found with the bacterial phylum Pseudomonadota (15.87%) followed by the fungi phylum Ascomycota (3.67%). There were two other bacterial phyla with Chloroflexota (1.60%) and Actinomycetota (0.42). The lowest percentage of β-glucosidase genes were detected in the plant phylum Tracheophyta (0.04%).

On the other hand, the other three soil locations showed only three different phyla with β-glucosidase genes. In the NJ soil the Pseudomonadota showed the highest numbers of β-glucosidase genes (17.48%), followed by Chloroflexota (2.00%) and Tracheophyta (0.51%). The highest percentages detected in all soils were found in the M soil with 18.12% of the β-glucosidase genes belonging to the Actinomycetota, 2.93% to the Ascomycota, and 0.29% to the Chloroflexota. The NY soil showed the lowest overall percentages of all soils with only 3.52% of the genes belonging to the Pseudomonadota, followed by Actinomycetota (0.58%), and Bacillota (0.02%). The Pseudomonadota were the number one phylum with β-glucosidase genes in three out of the four locations (NJ, NY, and D) while Actinomycetota sequences were dominant in the M soil. There was only one more phylum distributed in three different soils. This was the Chloroflexota (NJ, M, and D). Mold (M and D) and plant (NJ and D) sequences were found in two locations. Venn diagram analysis showed that the only category shared by all soils was the unclassified (Figure 1b).

A Jaccard similarity index value of 1 between soils indicated that β-glucosidase genes communities had the same composition at the phylum level. The closer to 1, the more similar they are. The values were NJ/NY (0.33), NJ/M (0.33), NJ/D (0.67), NY/M (0.33), NY/D (0.43) and M/D (0.67). The highest similarity was between M and D and NJ and D. The lowest was between NJ and NY and NJ and M. Average similarity values at the phylum level between all soils was 0.46.

### 3.2. β-Glucosidase Gene Soil Communities Based upon Bacteria, Fungi, and Plant Genera

Most β-glucosidase genes were unclassified DNA sequences that did not match any known genus. NY showed the highest percentage of unclassified sequences (95.62%), followed by M (84.44%), NJ (80.02%), and D (74.21%).

**Figure 2:**
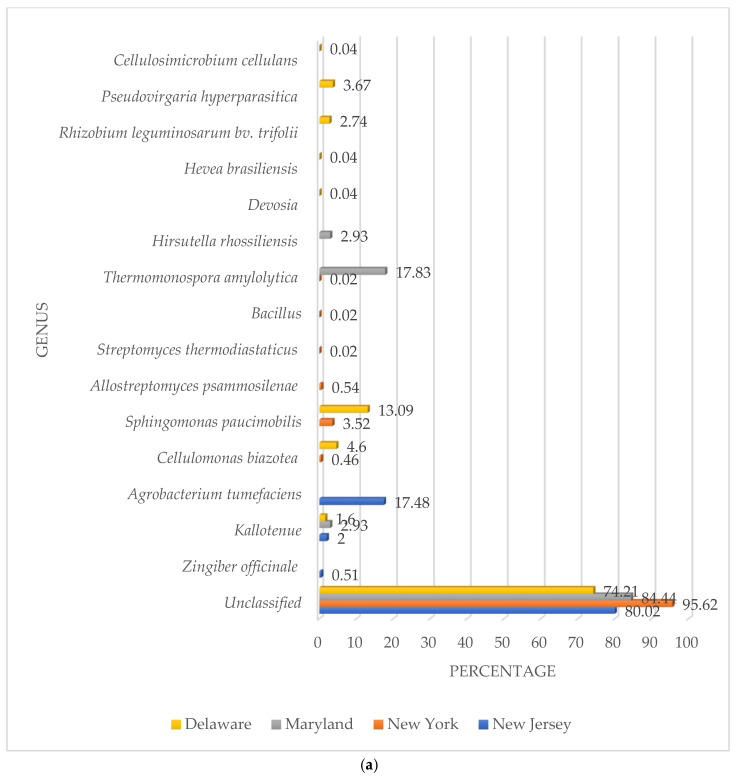

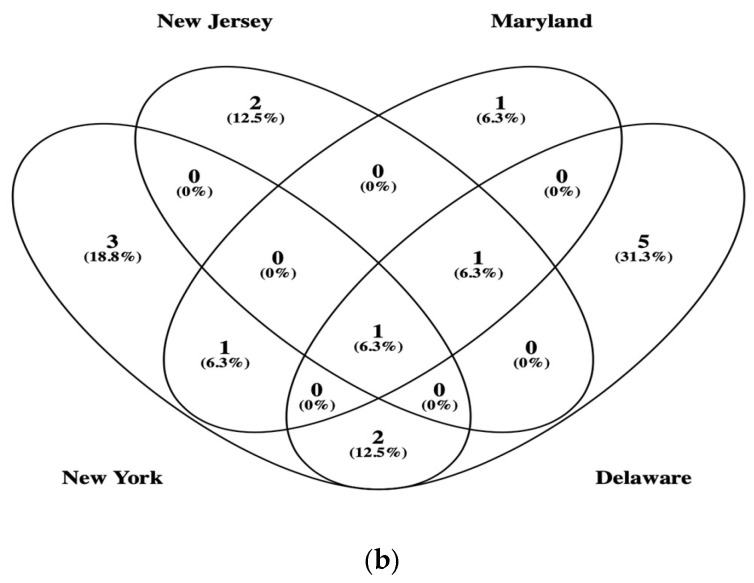
(**a**) Percentage of β-glucosidase genes by genus or species; (**b**) Venn diagram percentages of common species.

When identification was possible, a total of 15 types of β-glucosidase genes were detected in all four soils at the genus or species levels (Figure 2a). The highest numbers were detected in the D soil with 8. *S. paucimobilis* showed the highest numbers for β-glucosidase genes in D soil with 13.09%. Bacteria were the most dominant genera with *Rhizobium leguminosarum* bv. trifolii (2.74%), *C. biazotea* (4.60%), *Kallotenue* (1.60%), *Cellulosimicrobium cellulans* (0.40%), and *Devosia* sp. (0.40%). There was a fungus, *Pseudovirgaria hyperparasitica* (3.67%) and a plant species *Hevea brasiliensis* (0.04%) with β-glucosidase genes.

However, the less diverse community with β-glucosidase genes was found in the NJ soil. There were only three different types of populations with *A. tumefaciens* showing 17.48%, *Kallotenue* (2.00%), and the plant species *Zingiber officinale* (0.51%). The highest percentage of β-glucosidase genes in all soils was found in the M soil with 17.83% belonging to the bacterial species *T. amylolytica*, followed by the fungi *Hirsutella rhossiliensis* (2.93%), and the bacterial genus *Kallotenue* (2.93%).

The NY soil showed the second more diverse community with β-glucosidase genes, e.g., 6. The community was based upon only bacterial genera and species. The highest percentage belonged to the bacteria species *S. paucimobilis* (3.52%), followed by *Allostreptomyces psammosilenae* (0.54%), *C. biazotea* (0.46%), *T. amylolytica* (0.02%), *Bacillus* (0.02%), and *Streptomyces thermodiastaticus* (0.02%).

The only category shared by all soils was the unclassified (Figure 2b). However, the Chloroflexota genus *Kallotenue* was found in three soils (NJ, M, and D). *S. paucimobilis* was detected in two soils (NY and D) while *T. amylolytica* was present in M and D. A similarity value of 1 between soils indicated that β-glucosidase gene communities had the same composition at the genus or species level. The closer to 1, the more similar they are. The values were NJ/NY (0.10), NJ/M (0.33), NJ/D (0.18), NY/M (0.20), NY/D (0.23), and M/D (0.18). The highest similarity was found between NJ and M. The lowest was between NJ and NY. Average similarity values at the genus and species levels between soils was 0.20.

### 3.3. Phylogenetic Analysis of β-Glucosidase Gene Sequences

**Figure 3:**
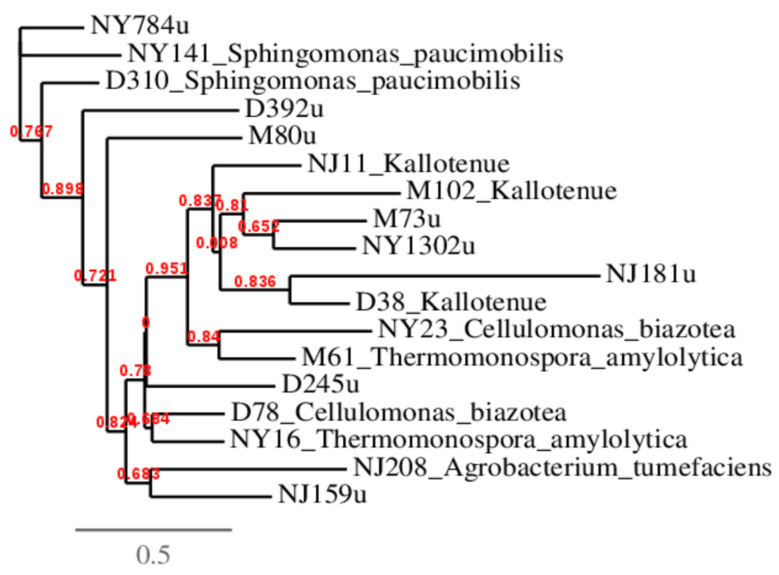
Phylogenetic analysis of β-glucosidase gene sequences. The branch length is proportional to the number of substitutions per site. D = Delaware; M = Maryland; NY = New York; NJ = New Jersey; u = unclassified. The values after state abbreviations represent the number of common sequences (100% similarity) detected. Red numbers represent bootstrap values.

Figure 3 shows the results for the phylogenetic analysis of unclassified sequences with identified bacterial β-glucosidase genes. The top two most abundant unclassified sequences found in each soil were compared to the known bacteria genera detected in this study. Results showed that most sequences clustered with the Pseudomonadota species *S. paucimobilis* and the Chloroflexota genus *Kallotenue*. The highest numbers of unclassified sequences of β-glucosidase genes detected in the soils from NY (1302u) clustered with *Kallotenue* detected in D and NJ soils while the second highest unclassified sequences also found in the soil from NY (784u) clustered with *S. paucimobilis* detected in D and NY soils. Other samples clustering with *Kallotenue* were NJ (181u) and M (73u). *S. paucimobilis* showed a closer phylogenetic relationship with D (392u) and M (80u).

## 4. Discussion

Biochemical reactions driven by microorganisms provide the foundation to recycle carbon compounds in the environment. Cellulose is the number one carbon source contributing to bacterial respiration in soil [1]. ASV sequences of the β-glucosidase gene detected in each soil habitat were clustered at 100% similarity. The majority of β-glucosidase gene sequences in all four soils analyzed showed no homology to any phyla or genera. This demonstrated the inability of the current databases to identify environmental β-glucosidase gene sequences and ascertain the genetic diversity in the studied soils. Similar results were reported by Jimenez et al. [15] and Pathan et al. [26] where high percentages of gene sequences were reported to be unclassified. Evidently, the great majority of taxa containing β-glucosidase gene sequences in the four soils analyzed in this study cannot be identified due to the known limitations of online databases. Therefore, there is a tremendous potential for discovery of novel enzymes for possible applications in the food, pharmaceutical, and biotechnology industries [28].

Even though GH1 and GH3 genes are found in most bacterial phyla, in this study, when identification was possible, the Pseudomonadota bacteria were the predominant phylum carrying GH1 β-glucosidase sequences [20]. Previous studies showed the Pseudomonadota to be dominant contributors of β-glucosidase genes in soils from different locations around the world [24,30,31,32,33,43]. Pseudomonadota are Gram-negative bacteria with the ability to carry on different metabolic processes such as carbon fixation, nitrogen fixation, cellulose biodegradation, etc. They can also grow under oligotrophic or copiotrophic environments.

The Pseudomonadota genera with the highest percentages overall in all four locations were *S. paucimobilis*. *S. paucimobilis* exhibited the highest percentages of all the genera detected in soil D and was also the number one population carrying β-glucosidase genes in the NY soil. Phylogenetic analysis of unclassified β-glucosidase genes from different soil locations also clustered with *S. paucimobilis*. *Sphingomonas* are known to be oligotrophic bacteria living under low nutrient concentration which are probably the conditions in the NY forest soil. Zeng et al. [32] reported that *Sphingomonas* demonstrated a significant association with β-glucosidase activity in forest and citrus soils. Abundance significantly declined with plantation age. However, it increased with the addition of organic carbon to citrus soils leading to the accumulation of SOC. Citrus plantation decreased the soil bacterial β-glucosidase gene abundance and SOC in comparison with forest soil. Moreno et al. [24] demonstrated that *Sphingomonas* was a predominant member of the β-glucosidase gene community in semiarid soils in Spain. The soil community structure was affected by the edaphic factors in soil. The presence of cover vegetation enhanced gene abundance and community diversity when compared to non-covered herbicide treated soils. *Sphingomonas* bacteria were also reported to be the most abundant β-glucosidase-producing microorganisms in ginseng soils in China [44].

The soil from D showed the highest diversity containing not only bacteria but also fungi and plant β-glucosidase genes. The second highest percentage of β-glucosidase genes were found in the Actinomycetota species *C. biazotea*. These bacteria are known cellulose degraders in soils enhancing the recycling of carbon [45]. In this study *C. biazotea* bacteria were also detected in the NY soil. Recent studies reported the presence of all three cellulases in *C. biazotea* [46]. However, the synergistic effect between endoglucanases, exoglucanases, and β-glucosidase activity appeared to be very weak based upon the inefficient performance in filter paper hydrolysis.

The presence of β-glucosidase genes of fungal origin was detected in soil D. BLAST analysis identified the sequences belonging to *P. hyperparasitica* which is a known parasite (mycoparasite) of rust fungi, specifically different species of *Frommeella*, *Pucciniastrum*, and *Phragmidium* [47]. Mold sequences were also found in soil M. The β-glucosidase sequences were identified to belong to *H. rhossiliensis*. These molds are nematophagous fungi [48]. They parasitize nematodes in soil by penetrating their body, resulting in the destruction of the worm by the fungus. Because of this they can be used as biocontrol agents against nematodes. Both *P. hyperparasitica* and *H. rhossiliensis* belong to the phylum Ascomycota which are widely distributed in soils around the world and provide significant activity in the production of glucose from cellobiose [16,25,26,27,32,33]. In some soils the contribution of fungi β-glucosidase genes was found to be more significant than bacteria during the degradation of cellobiose to glucose [27]. Fungi genes were directly correlated with enzyme activity. However, that was not the case with bacterial β-glucosidase genes. However, in this study, most communities were dominated by bacterial genes. Fungi β-glucosidases are mostly present in saprophytic fungi which are responsible for the decomposition of cellulose contributing to the maintenance of soil quality and the release of nutrients such as glucose into the soils. They are extremely sensitive to glucose inhibition. Fungi such as *Aspergillus* and *Trichoderma* are the major sources of β-glucosidase for commercial production because of the high number of enzymes produced and the relative tolerance to high concentrations of glucose. Nevertheless, other factors such as narrow pH ranges and low thermal stability severely limited their application to several industrial processes. Other fungi known to be high producers of β-glucosidase are *Coniophora puteana*, *Chaetomella* spp., and *Microdochium*. Yeasts such as *Saccharomyces cerevisiae* and *Pichia* also were shown to be good producers of these enzymes.

Very low abundance values of β-glucosidase genes of plant origin were detected in soils D and NJ. These gene sequences showed the closest homology to *H. brasiliensis* (rubber tree) in D and *Z. officinale* (ginger) in NJ. GH1 β-glucosidase genes are responsible for several developmental and stress responses in plants [49,50]. For instance, they are involved in the plant defense against pathogens, phytohormone regulations, lignification, and cell wall degradation. In *Brassica rapa* and *Arabidopsis*, stress responses are regulated by the action of β-glucosidase activity where the cleavage of glucose-conjugated abscisic acid (ABA-GE) by the action of β-glucosidase releases abscisic acid into the cell triggering drought tolerance by the cells. Pollen development is also affected by the action of these enzymes. Mutations in β-glucosidase genes led to defective pollen wall formation [49]. When plants get injured, scopolin (a glucoside of scopoletin) is released into the cytoplasm from the vacuoles and then it is hydrolyzed by β-glucosidase enzymes present in the cytoplasm resulting in the production of scopoletin and glucose. This compound can act as a defense mechanism against pathogens. In *Oryza sativa* spp. *japonica* (rice), most of the β-glucosidase genes are clustered on the same chromosomes [50]. For instance, Gibberellin glucosides (GG) are plant hormones with the addition of glucose molecules which provides a way to store the Gibberellins (G) in plant cells. They are very important hormones related to seed germination and stem elongation. The action of β-glucosidase enzymes by hydrolyzing GG released G into the cells promoting plant growth. Another example of the importance of β-glucosidase activity in plants is their role in the hydrolysis of SAβ-glucoside (SAG) to glucose and salicylic acid (SA). This hydrolysis is triggered during an infection where SA will be involved in the defense against a pathogen and stresses affecting the plant cell [51]. On the other hand, glucose release can influence developmental processes such as root and stem growth.

The NJ and M soils showed the lowest diversity of β-glucosidase sequences with only three different types of populations. In the NJ soil, the predominant bacteria with β-glucosidase genes were the Pseudomonadota species *A. tumefaciens* while in the M soil, it was the Actinomycetota species *T. amylolytica* formerly known as *Actinomadura amylolytica*. *A. tumefaciens* is commonly found in soils where in some situations, it is responsible for the plant disease crown gall, which triggers the formation of tumors in tissues [44,51,52,53]. This is performed by the incorporation of the Ti plasmid (T-DNA) into the plant genome [53]. In addition to contributing to the biodegradation of cellobiose to glucose, β-glucosidase activity also regulated pathogenic genes expression by releasing salicylic acid (SA) from the storage form inside of the plant, e.g., SAG [54]. SA is released by the action of β-glucosidase activity after the infection by *A. tumefaciens* is completed. SA decreased the expression of virulence genes responsible for the initial infection and tumor formation.

*T. amylolytica* is a thermophilic cellulolytic bacteria commonly inhabiting soils that are exposed to high temperatures and also compost material [55]. However, thermophilic bacteria are also found in temperate soils [56,57]. They are the source of a remarkable number of thermostable cellulases currently used to optimize cellulose conversion to glucose [58]. Furthermore, extracellular enzymes from thermophilic bacteria showed a longer stability in soils under different temperate and water availability conditions [57].

There was only one type of β-glucosidase genes distributed in three out of the four soil locations. Soils NJ, M, and D showed the presence of β-glucosidase genes associated with the Chloroflexota genus *Kallotenue* [59]. Furthermore, unclassified β-glucosidase genes clustered with *Kallotenue* genes at different soil locations. *Kallotenue* was first reported to be isolated from a lignocellulosic enrichment in a geothermal spring in the state of Nevada, USA. They are cellulolytic thermophilic bacteria with the ability to produce different types of cellulases [60]. They were also reported to be dominant members of the bacterial community in oligotrophic arid bulk soil samples from the Cuatro Cienegas Basin in the Chihuahuan Desert in Mexico [61]. Bacteria such as *Kallotenue* can retain humidity and extend through the soil matrix due to the filamentous nature enhancing the acquisition of nutrients. Cyanobacterial crust (Cc) collected from desert soil samples were exposed to extreme conditions in the stratosphere, high ultraviolet light (UV) and low temperature. *Kallotenue* was one of the most tolerant genera to UV and low temperature. The relative abundance of *Kallotenue* increased along with the activity of β-glucosidase in Cc [62]. Microbial degradation of cellulose and chitin was one of the mechanisms by which Cc resisted stratospheric conditions. Other studies showed that after mature biofilm was developed on membrane surfaces in a membrane distillation process, *Kallotenue* emerged as a major member of the bacterial community due to the high concentration of SOC in the membrane distillation using treated effluent from a wastewater treatment plant [63]. Biofilm accumulation on membranes during treatment due to the accumulation of organic carbon and extracellular polymeric substances (EPS) led to the emerging of *Kallotenue* during the last thermophilic stages of the treatment. Evidently, the metabolic diversity and tolerance of different environmental fluctuations allowed bacteria belonging to the genus *Kallotenue* to be widely distributed in three out of the four soils analyzed.

Soil properties such as pH, SOC, temperature, soil management, and other edaphic factors were not analyzed in this study which might be affecting the distribution and community diversity of taxa containing β-glucosidase genes. SOC, pH, and land management were reported to be very important factors affecting β-glucosidase activity and community diversity in soils [16,27,31,32]. Future studies will determine the effect of different edaphic factors on the distribution and community structure of taxa with β-glucosidase genes. The use of PCR and specific primer pairs along with next generation sequence can introduce some bias by selecting specific genotypes and underestimating low abundance populations or other GH 1 sequences that might not be detected. Nevertheless, the number of β-glucosidase gene sequences analyzed were significantly higher than previous studies reported in soils from the Northeastern USA where the number of clones analyzed by Sanger sequencing were extremely low [15,31]. The use of multiple primer pairs can also optimize the numbers of β-glucosidase gene sequences detected. Similar studies were performed to analyze the diversity of cellulases genes, e.g., GH48, in biogas fermenters [19]. In that study 17 primers were used in different combinations, allowing the detection and characterization of a wider spectrum of cellulolytic communities carrying GH48 genes. Future studies will evaluate the use of multiple primer pairs to ascertain the presence of β-glucosidase genes in soils. GH1 genes in the studied soils were mostly related to bacteria with low percentages of fungi and plant genes. However, this study provided a better understanding of the undiscovered diversity of taxa with the capability to carry β-glucosidase genes indicating the possibility to ascertain with greater depth and resolution the community structure of these important genes responsible for the biogeochemical cycle of cellulose in soils. Nevertheless, the statistical robustness and representative nature of these results might not express the wide variety of GH1 gene sequences in soils due to the limited representation of the four locations (spatial variability) and the insufficient number of replicate soil samples analyzed which can lead to a reduced statistical analysis. Because of the heterogenous nature of soil, gene distribution can be affected by factors such as soil texture, plant growth, pH, SOC, moisture, etc. This situation can lead to a misunderstanding of the results to be representative of a broader pattern instead of a specific localized variation. In this study the findings represent only localized patterns of GH1 genes to specific soil locations and do not intend to propose a wider pattern of gene distribution across all four locations analyzed.

Furthermore, the genetic potential present in the tested soils for the biodegradation of cellobiose to glucose by the presence of β-glucosidase genes is not an indication that these genes are actually active and expressed and that these communities are actually degrading cellulose. However, gene expression can be analyzed by using RNA sequencing (RNA-seq) or quantitative PCR (qPCR). Verification of gene expression will confirm community functionality and viability. In a previous study, the gene expression of β-glucosidase genes was analyzed during different stages of composting [64]. They concluded that glucose-tolerant and glucose-intolerant β-glucosidase genes were differentially expressed during composting when cellulose degradation conditions were changing during the process as thermophilic and cooling stages were taking place. Most GH1 genes were related to bacteria belonging to the phyla Pseudomonadota and Actinomycetota. However, the metatranscriptome data was dominated by Pseudomonadota and Bacteroidota. However, some of the sequences belonged to unclassified bacteria. The transcription of β-glucosidase genes was lower in the cooling phases compared to the thermophilic phases. Gene expression of β-glucosidase genes was studied in summer and late winter in soils located in the Bohemian Forest Mountain range in Central Europe [26]. Results indicated that up to 70% of the transcripts were unclassified at the phylum level. When identification was possible, most transcripts belonged to bacteria from the phylum Bacillota. In fungi, most transcripts were found to be related to the phylum Basidiomycota. The transcript pool of β-glucosidase genes was less diverse than the gene pool indicating that a fraction of the microbial community was actively metabolizing cellobiose to glucose. 

The presence of novel and unclassified GH1 genes in all soils provided a potential source for the possible identification of new enzymes [65]. The original purpose of the study was to identify GH1 genes in soils to understand the community structure and diversity of microorganisms with β-glucosidase genes. However, most GH1 genes detected did not match any known organisms providing an unexplored source for unlimited genetic and biochemical activities. For instance, β-glucosidases belonging to the GH1 family are more resistant to inhibition by glucose which is the end product of the biodegradation of cellobiose [26,65]. Industrial processes such as biofuel production where cellulose degradation is converted to ethanol can be optimized by the use of enzymes with high glucose tolerance and chemical stability under extreme conditions, e.g., high temperature and extreme pH. Furthermore, novel β-glucosidases can also be applied to the synthesis of new pharmaceutical drugs by chemical structure modification. The possibility of the discovery of unique enzyme properties can open other possible applications to food, beverage, and textile manufacturing.

## Figures and Tables

**Table 1:** Accession numbers for identified β-glucosidase genes used in phylogenetic analysis.

Genus/Species	Accession Number
*Agrobacterium tumefaciens*	KU512832.1
*Cellulomonas biazotea*	JF727823.1
*Kallotenue*	OR724869.1
*Sphingomonas paucimobilis*	MW804636.1
*Thermomonospora amylolytica*	MH974517.1

## Data Availability

The data will be available upon request to the corresponding author.
